# The impact of lifetime substance use on psychiatric comorbidities and treatment seeking in patients with alcohol use disorders

**DOI:** 10.1038/s41598-024-65028-x

**Published:** 2024-06-20

**Authors:** Piotr Engelgardt, Maciej Krzyżanowski, Małgorzata Borkowska-Sztachańska, Agnieszka Wasilewska, Michał Ciucias

**Affiliations:** 1 Department of Pathomorphology and Forensic Medicine, University of Warmia and Mazury, Olsztyn, Poland; 2 Department of Psychiatry, University of Warmia and Mazury, Olsztyn, Poland; 3 Department of Anatomy, University of Warmia and Mazury, Olsztyn, Poland

**Keywords:** Alcohol use disorder, Psychiatric comorbidity, Drug use disorders, Epidemiology, Health care, Risk factors

## Abstract

It is well-recognized that individuals with alcohol-related disorders often use other psychoactive substances; however, systematic research on this topic remains limited. The primary objective was to determine the prevalence of lifetime psychoactive substance use and describe the dependence between concurrent use of alcohol and other drugs on psychiatric comorbidities in the analyzed group. The secondary aim was to try to assess the frequency of seeking psychiatric treatment between individuals declaring the concurrent use of alcohol with other drugs and those declaring the use only alcohol. The study was designed as a retrospective cross-sectional analysis based on discharge reports from psychiatric patients admitted to the Regional Psychiatric Hospital in Olsztyn, Poland. 1015 cases were included and analyzed in the study. Data for the study were collected in specially designed monitoring cards from discharge reports including data from psychiatric examinations, especially anamnesis. The percentage of people declaring lifetime use of psychoactive substances was 17.6%. 2.8% of them were diagnosed with substance-related disorders (F11–19 according to ICD-10). The most frequently declared use was cannabis, followed by amphetamine-type substances, benzodiazepines and new psychoactive substances. In the group of people declaring the lifetime use of psychoactive substances, 13.4% were additionally diagnosed with mental disorders. It was, consequently, 8% in the group of people denying the lifetime use of psychoactive substances. People declaring lifetime use of psychoactive substances were significantly more likely to seek psychiatric treatment, i.e. they were admitted significantly more often on an emergency admission than on an elective one, these people were significantly more likely to have undergone psychiatric treatment in the past and were more often hospitalized in our center during the research period. People who concurrently use alcohol with other drugs significantly more often have psychiatric comorbidity than people who deny the use of other drugs. That group also visibly more often seeks psychiatric treatment than patients who deny taking psychoactive substances.

## Introduction

Alcohol is one of the most commonly used drugs, with up to 290 million people diagnosed with an alcohol use disorder (AUD) worldwide^1^. This translates to 29.5 million people in the US only. This includes 8 million people with an additional diagnosis of substance use disorder (SUD)^2^. In the literature, the term DUD (drug use disorder) is sometimes used interchangeably with SUD. In Poland, according to EZOP’s “Comprehensive Study of the State of Mental Health of Society and its Determinants” performed in 2018–2021, the number of people with AUD is estimated at approximately 582 thousand^3^.

Alcohol is frequently used with other substances. The National Epidemiologic Survey on Alcohol and Related Conditions (NESARC) conducted in 2012–2013 indicated that 46.5% of the individuals with AUD also had one or more concurrent substance use disorders, specifically, 31.5% with comorbid nicotine use disorder, 3.2% with comorbid cannabis use disorder, and 6.1% with a comorbid SUD involving other drugs such as opioids or stimulants^4^.

There is a significant link between the use of cannabis and alcohol early in life and the ongoing simultaneous use of both substances in cases with dual diagnoses, as opposed to cases of AUD or cannabis use disorder (CUD) alone^5–7^. Modern psychiatric epidemiology indicates that the developmental paths leading to alcohol use disorder start before the onset of problematic alcohol consumption^8^.

There is a high percentage of mental disorders in people with AUD, depending on the research methodology. Thus, in a study conducted by Morandi et al.^9^, 10.5% of patients, who received treatment in an alcohol center, had a dual diagnosis (with a higher prevalence in women).

Historically, 40.6% of men and 47.1% of women with alcohol use disorder have also had a lifetime substance use disorder^8^.

When examining groups of people with particular psychiatric conditions, a higher percentage of patients in these groups meet the criteria for AUD. For instance, the rate is approximately 30% for individuals with mood disorders, up to 50% for those with anxiety disorders, ranging from 20 to 65% for schizophrenia spectrum disorders, and potentially exceeding 80% for bipolar disorders^10,11^.

Similarly, major depressive disorder is the most common psychiatric condition that co-occurs with AUD. Specifically, individuals with AUD, compared to those without AUD, were 2.3 times more likely to have experienced major depressive disorder in the past year, and 1.7 times more likely to have had dysthymia during that period^12^.

However, when assessing lifetime psychiatric comorbidity in people diagnosed with AUD, Fein’s study shows that it affects more than ¾ of people with such disorder^13^.

Polydrug use that includes alcohol is associated with additional comorbidities, including a higher prevalence of mood disorders, anxiety disorders, more intense drinking, and more intense drug consumption and drug craving. The results of the study by Saha et al. underscored the high prevalence, the high prevalence, increased severity and more severe psychopathology of concurrent alcohol use with other drugs, and the co-occurrence of AUD and DUD compared to those who used only alcohol or who were only diagnosed with an alcohol use disorder^4^. This could be explained as follows, patients with psychiatric comorbidity or polysubstance use disorders because of the functional impairments and distress, are more likely to seek treatment than patients with a single disorder^14^. Although there is literature on the co-occurrence of AUD–DUD in the general population^2,4,14^ and the concurrent use of alcohol and other psychoactive substances among people with AUD^5–8,10–12^, little is known about the difference in the frequency of seeking psychiatric treatment between groups of people declaring the concurrent use of alcohol with other drugs, and people declaring the use of alcohol only.

## Material and methods

### Objectives

The primary objective of this study was to determine the prevalence of lifetime psychoactive substance (PS) use and to explore the association between the concurrent use of alcohol and other drugs and psychiatric comorbidities among individuals diagnosed with alcohol-related disorder (classified as F10 according to the ICD-10).

Additionally, this study aimed to compare the frequency of seeking psychiatric treatment between two groups: those who reported concurrent use of alcohol and other drugs, and those who reported using only alcohol.

### Study design and data collection

The study was designed as a retrospective cross-sectional analysis based on discharge reports from psychiatric patients admitted to the Regional Psychiatric Hospital in Olsztyn, Poland. The analysis included adult patients (18–79 years old) diagnosed with mental and behavioral disorders due to the use of alcohol (F10 according to the ICD-10), who were admitted to the hospital between September 1, 2018, and September 30, 2019. Patients were identified through the hospital’s medical database. The entire medical documentation of the patients was analyzed, including medical records, discharge summaries and consultation notes. Thus, the reliability of reported outcomes was dependent on the clinical evaluations and patient self-reports. All analyzed data were recorded in an Excel spreadsheet. Key patient information presented in the documentation encompassed age, gender, psychiatric diagnosis according to ICD-10, mode of hospital admission (emergency or elective), taking psychoactive substances (at least once in a lifetime, taken for non-therapeutic reasons) and history of psychoactive substance use (at least once in a lifetime, for non-therapeutic purposes), including the types of substances used. Psychoactive substances were defined as any that are used for intoxication or other mind-altering effects. On the other hand, information regarding cigarette usage was excluded from this study.

The second step was carried out to assess the relationship between age, sex, and self-reported use of psychoactive substances. Subsequently, we checked whether there was a relationship between psychiatric comorbidities and self-reported use of psychoactive substances. In these steps, the analysis regarded all categories of psychoactive substances, and the most commonly reported specific substance usage was further explored.

In the final step, we aimed to explore the potential linkage between the reported use of psychoactive substances and the need to seek psychiatric treatment. This was evaluated by considering the frequency of hospital admissions during the study period (verified with PESEL numbers) and the mode of these admissions (emergency or elective). Additionally, we used anamnesis data related to previous psychiatric interventions, accounting for both outpatient and inpatient care. The PESEL is an identification number given to every Polish citizen and foreign resident in Poland, facilitating the verification of an individual’s identity and their administrative and legal status. Emergency admissions were defined as cases where patients exhibited symptoms of mental disorders that posed a threat to their lives, yet without immediate somatic complications. Patients presenting acute, life-threatening symptoms due to intoxication were initially treated in the Emergency Ward, and then transferred to appropriate departments, such as Intensive Care Unit, Toxicology Department or others, depending on their specific health needs and overall condition.

### Data analysis

All data were transferred into an IBM SPSS Statistics Package, version 27 (IBM Corp., Armonk, NY) and coded. The significance threshold for all tests was determined at the level of *α* < 0.05. Cases with incomplete medical reports were identified and excluded from further analyses. The remaining cases were included in the study.

Data were expressed as frequencies; additionally, percentage values were provided. Individuals’ age data were arranged into the following age groups: 18–30, 31–40, 41–50, 51–60, and > 60 years.

Initially, descriptive analyses were conducted to examine the distribution of age groups, sex, and total psychoactive substance users, followed by further investigation of the frequency of specific substance use. Specific substances included cannabis, amphetamine-type substances (ATS), new psychoactive substances (NPS), benzodiazepines (BDZ), opioids (OPI), and cocaine (COC). The ATS group consisted of amphetamine and its derivatives: MDMA and methamphetamine. Tramadol, when taken for non-therapeutic reasons, was classified as an opioid, and included in the OPI group*.*

Pearson Chi-squared tests were conducted to assess the significance of differences in sex and age, as well as to determine the distinctions between single and multiple PS users.

In the next step, cross tabs were obtained for age groups and PS use (both overall and specific substances), accounting also for gender differences (Table 1). The impact of specific age groups on PS use was evaluated using the bivariate logistic regression (Table 2). In this analysis, each category of PS use was treated as a separate dependent variable, and individual age groups were introduced sequentially as independent predictors within the regression model.

**Table 1:** The types of psychoactive substances which were declared in anamnesis according to age group and sex.

Age group	Total (N = 1015)	PS (N = 179)	Cannabis (N = 115)	ATS (N = 65)	BDZ (N = 30)	NPS (N = 29)	OPI (N = 24)	COC (N = 16)
	N (%) Male N (%) Female N (%)	ID + N (%) Male ID + N (%) Female ID + N (%)	N (%) Male N (%) Female N (%)	N (%) Male N (%) Female N (%)	N (%) Male N (%) Female N (%)	N (%) Male N (%) Female N (%)	N (%) Male N (%) Female N (%)	N (%) Male N (%) Female N (%)
All	1015 (100%) 827 (81.5%) 188 (19.5%)	179 (17.6%) 153 (15.1%) 26 (2.6%)	115 (11.3%) 105 (13.4%) 10 (1%)	65 (6.4%) 58 (5.7%) 7 (1.1%)	30 (3%) 19 (1.9%) 11 (1.1%)	29 (2.9%) 26 (2.6%) 3 (0.3%)	24 (2.4%) 21 (2.1%) 3 (0.3%)	16 (1.6%) 16 (1.6%) 0 (0%)
18–30	103 (100%) 84 (81.6%) 19 (18.4%)	47 (45.6%) 39 (37.9%) 8 (7.8%)	38 (36.9%) 32 (31.1%) 6 (5.8%)	21 (20.4%) 18 (17.5%) 3 (2.9%)	4 (3.9%) 4 (3.9%) 0 (0%)	15 (14.6%) 13 (12.6%) 2 (1.9%)	3 (2.9%) 3 (2.9%) 0 (0%)	5 (4.9%) 5 (4.9%) 0 (0%)
31–40	272 (100%) 226 (83.1%) 46 (16.9%)	81 (29.8%) 74 (27.2%) 7 (2.6%)	54 (19.9%) 50 (18.4%) 4 (1.5%)	36 (13.2%) 34 (12.5%) 2 (0.7%)	11 (4%) 9 (3.3%) 2 (0.7%)	13 (4.8%) 13 (4.8%) 0 (0%)	12 (4.4%) 10 (3.7%) 2 (0.7%)	8 (2.9%) 8 (2.9%) 0 (0%)
41–50	244 (100%) 200 (82%) 44 (18%)	32 (13.1%) 27 (11.1%) 5 (2%)	17 (7%) 17 (7%) 0 (0%)	7 (2.9%) 5 (2%) 2 (0.8%)	5 (2%) 2 (0.8%) 3 (1.2%)	1 (0.4%) 1 (0.4%) 0 (0%)	7 (2%) 6 (2.46%) 1 (0.4%)	3 (1.2%) 3 (1.2%) 0 (0%)
51–60	256 (100%) 208 (81.3%) 48 (18.8%)	10 (3.9%) 7 (2.7%) 3 (1.2%)	4 (1.6%) 4 (1.6%) 0 (0%)	0 (0%) 0 (0%) 0 (0%)	5 (2%) 2 (0.8%) 3 (1.2%)	0 (0%) 0 (0%) 0 (0%)	1 (0.4%) 1 (0.4%) 0 (0%)	0 (0%) 0 (0%) 0 (0%)
> 60	140 (100%) 109 (77.9%) 31 (22.1%)	9 (6.4%) 6 (4.3%) 3 (2.1%)	2 (1.4%) 2 (1.4%) 0 (0%)	1 (0.7%) 1 (0.7%) 0 (0%)	5 (3.6%) 2 (1.4%) 3 (2.1%)	0 (0%) 0 (0%) 0 (0%)	1 (0.7%) 1 (0.7%) 0 (0%)	0 (0%) 0 (0%) 0 (0%)

**Table 2:** Results of bivariate logistic regression.

Age group	F			M		
	*p*	*Exp*(*B*)	*C.I. l–h*	*p*	*Exp*(*B*)	*C.I. l–h*
*PS*						
18–30	0.08	3.51	1.39–8.85	< 0.001	4.89	3.08–7.8
31–40	0.66	0.83	0.37–1.89	< 0.001	3.17	2.25–4.48
41–50	0.27	0.59	0.23–1.51	0.09	0.68	0.44–1.06
51–60	0.05	0.30	0.09–0.98	< 0.001	0.13	0.06–0.28
> 60	0.25	0.49	0.15–1.64	< 0.001	0.25	0.11–0.57
*ATS*						
18–30	0.11	2.82	0.8–9.95	< 0.001	5.13	2.82–9.33
31–40	0.58	0.67	0.16–2.81	< 0.001	4.18	2.5–7
41–50	0.63	0.70	0.17–2.95	0.02	0.33	0.13–0.83
51–60	1.00	0	0–0	1.00	0.00	0–0
> 60	1.00	0	0–0	0.04	0.12	0.02–0.9
*Cannabis*						
18–30	0.009	3.76	1.4–10.08	< 0.001	6.29	3.83–10.31
31–40	0.57	0.74	0.26–2.09	< 0.001	3.16	2.11–4.74
41–50	1.00	0	0–0	0.16	0.68	0.4–1.17
51–60	1.00	0	0–0	< 0.001	0.12	0.05–0.34
> 60	1.00	0	0–0	0.01	0.13	0.03–0.54
*BDZ*						
18–30	0.99	0	0–0	0.31	1.74	0.59–5.11
31–40	0.57	1.53	0.35–6.62	0.31	1.52	0.69–3.36
41–50	0.14	2.56	0.75–8.78	0.09	0.28	0.07–1.2
51–60	0.18	2.32	0.68–7.94	0.08	0.27	0.06–1.14
> 60	0.04	3.80	1.09–13.26	0.47	0.59	0.14–2.49
*NPS*						
18–30	0.06	4.22	0.93–19.19	< 0.001	10.47	4.84–22.46
31–40	1.00	0	0–0	0.01	2.95	1.4–6.23
41–50	1.00	0	0–0	0.06	0.14	0.02–1.04
51–60	1.00	0	0–0	1.00	0	0–0
> 60	1.00	0	0–0	1.00	0	0–0
*OPI*						
18–30	0.99	0	0–0	0.45	1.61	0.47–5.5
31–40	0.37	1.95	0.44–8.58	0.03	2.56	1.23–5.85
41–50	0.99	0.95	0.12–7.26	0.51	1.37	0.53–3.5
51–60	0.99	0	0–0	0.08	0.16	0.02–1.23
> 60	0.99	0	0–0	0.31	0.35	0.05–2.66
*COC*						
18–30	0.99	0	0–0	0.003	5.29	1.8–15.61
31–40	0.99	0	0–0	0.012	3.58	1.33–9.65
41–50	0.99	0	0–0	0.92	0.94	0.26–3.33
51–60	0.99	0	0–0	0.99	0	0–0
> 60	0.99	0	0–0	0.99	0	0–0

Associations of age group and sex with PS use. The regression model was run for each PS type as a dependent variable, separately. Age group variables were used as predictors and entered sequentially into the regression model.

Significant age groups were then included concurrently in a multivariable regression model (Table 3).

**Table 3:** Results of multivariable logistic regression.

Age group	*p*	*Exp*(*B*)	*C.I. l–h*
*Females*			
PS			
51–60	0.045	0.31	0.09–0.98
THC			
18–30	0.009	3.76	1.4–10.08
BDZ			
> 60	0.04	3.80	1.09–13.26
*Males*			
PS			
18–30	< 0.001	5.48	3.26–9.2
31–40	< 0.001	3.07	2.1–4.6
51–60	< 0.001	0.22	0.1–0.49
> 60	0.03	0.37	0.15–0.88
ATS			
21–30	< 0.001	15.16	6.09–37.7
31–40	< 0.001	9.84	4.29–22.61
41–50	0.55	1.425	0.45–4.55
> 60	0.54	0.515	0.06–4.23
Cannabis			
21–30	< 0.001	8.2	4.56–14.82
31–40	< 0.001	3.8	2.3–6.272
51–60	0.014	0.26	0.09–0.76
> 60	0.061	0.25	0.06–1.07
NPS			
18–30	< 0.001	42.84	11.92–153.92
31–40	< 0.001	14.28	4.03–50.58
OPI			
31–40	0.03	2.56	1.23–5.85
COC			
18–30	< 0.001	14.81	3.47–63.14
31–40	0.002	8.58	2.26–32.6

Associations of age group and sex with PS use. A regression model was run for each PS type as the dependent variable, separately. However, significant (as found in the bivariate regression) age groups were entered into the model concurrently.

Subsequently, cross-tabs were obtained to explore the prevalence of comorbid psychiatric disorders across PS use (Table 4). This step was followed by bivariate and multivariable logistic regression, to examine the relationship between these disorders occurrence and categories of PS use.

**Table 4:** Additional diagnosis (excluding F10–F19).

	Total (N = 1015)	No PS (N = 836)		PS (N = 179)		Cannabis (N = 115)		ATS (N = 65)		BDZ (N = 30)		NPS (N = 29)		OPI (N = 24)		COC (N = 16)	
	N	N	% of PS-	N	% of PS	N	% of THC	N	% of ATS	N	% of BDZ	N	% of NPS	N	% of OPI	N	% of COC
F00–F99 (without F10–F19)	91	67	8%	24	13.4%	13	11.3%	5	7.7%	9	30%	2	6.9%	2	8.3%	3	18.8%
F00–F09 organic including symptomatic mental disorders	21	16	1.9%	4	2.2%	1	0.8%	0	0	3	10%	0	0	0	0%	1	6.3%
F20–F29 schizophrenia schizotypal and delusional disorders	22	15	1.8%	7	3.9%	7	6.1%	3	4.6%	1	3.3%	2	6.9%	1	4.2%	2	12.5%
F30–F39 mood [affective] disorders	15	12	1.4%	3	1.7%	0	0	0	0	2	6.7%	0	0	0	0%	1	6.3%
F40–F49 Neurotic stress-related and somatoform disorders	16	11	1.3%	5	2.8%	3	2.6%	1	1.5%	2	6.7%	0	0	0	0%	0	0%
F50–F59 behavioral syndromes associated with physiological disturbances and physical factors	1	1	0.1%	0	0	0	0	0	0	0	0	0	0	0	0%	0	0%
F60–F69 disorders of adult personality and behavior	7	7	0	0	0	0	0	0	0	0	0	0	0	0	0%	0	0%
F70–F79 mental retardation	11	6	0.7%	5	2.8%	2	1.7%	1	1.5%	1	3.3%	0	0	1	4.2%	0	0%
F80–F89 disorders of psychological development	0	0	0	0	0	0	0	0	0	0	0	0	0	0	0%	0	0%
F90–F99 behavioral and emotional disorders with onset usually occurring in childhood and adolescence	1	1	0.1%	0	0	0	0	0	0	0	0	0	0	0	0%	0	0%

Numbers represent frequencies. Numbers in parentheses represent percentage values of the given type total N.

At this stage, comorbid psychiatric disorders were treated as dependent variables in the regression models, with each disorder group analyzed separately. PS overall use, as well as the four most frequently used substances, were individually entered as predictors in the bivariate analysis (Table 5). Significant predictors were subsequently included in a multivariable regression model to assess their combined impact (Table 6).

**Table 5:** Results of bivariate logistic regression. Risk of disorders occurrence according to PS use.

	*p*	*Exp*(*B*)	*C.I l*–*C.I. h*
*PS*			
F00–F99	0.023	1.777	1.08–2.92
F00–F09	0.46	1.473	0.53–4.1
F20–F29	0.085	2.228	0.9–5.55
F30–F39	0.81	1.1	0.33–4.2
F40–F49	0.16	2.16	0.79–6.28
F50–F59	0.996	0	0
F60–F69	0.996	0	0
F70–F79	0.024	3.975	1.2–13.17
F90–F99	0.996	0	0
*Cannabis*			
F00–F99	0.353	1.343	0.72–2.5
F00–F09	0.355	0.386	0.05–2.9
F20–F29	0.004	3.824	1.53–9.59
F30–F39	0.996	0	0
F40–F49	0.352	1.82	0.51–6.51
F50–F59	0.997	0	0
F60–F69	0.997	0	0
F70–F79	0.477	1.752	0.37–8.21
F90–F99	0.997	0	0
*ATS*			
F00–F99	0.711	0.84	0.33–2.14
F00–F09	0.997	0	0
F20–F29	0.174	2.37	0.68–8.23
F30–F39	0.998	0	0
F40–F49	0.98	0.97	0.13–7.5
F50–F59	0.998	0	0
F60–F69	0.997	0	0
F70–F79	0.716	1.47	0.19–11.66
F90–F99	0.998	0	0
*BDZ*			
F00–F99	< 0.001	4.72	2.09–10.64
F00–F09	0.006	5.97	1.66–21.49
F20–F29	0.659	1.583	0.21–12.17
F30–F39	0.032	5.341	1.15–24.8
F40–F49	0.04	4.95	1.07–22.84
F50–F59	0.998	0	0
F60–F69	0.998	0	0
F70–F79	0.255	3.362	0.42–27.14
F90–F99	0.998	0	0
*NPS*			
F00–F99	0.693	0.747	0.18–3.19
F00–F09	0.998	0	0
F20–F29	0.096	3.578	0.8–16.08
F30–F39	0.998	0	0
F40–F49	0.998	0	0
F50–F59	0.998	0	0
F60–F69	0.998	0	0
F70–F79	0.998	0	0
F90–F99	0.998	0	0
*OPI*			
F00–F99	0.913	0.92	0.23–4.21
F00–F09	0.998	0	0
F20–F29	0.51	2.01	0.26–17.6
F30–F39	0.99	0	0
F40–F49	0.99	0	0
F50–F59	1	0	0
F60–F69	0.99	0	0
F70–F79	0.18	4.26	0.55–34.7
F90–F99	1	0	0
*COC*			
F00–F99	0.26	3.26	0.41–25.9
F00–F09	0.99	0	0
F20–F29	0.014	6.993	1.5–32.83
F30–F39	0.15	4.7	0.58–37.9
F40–F49	0.99	0	0
F50–F59	0.99	0	0
F60–F69	0.99	0	0
F70–F79	0.99	0	0
F90–F99	0.99	0	0

Bivariate logistic regression results indicated variations in the risk of disorder occurrence based on psychoactive substance (PS) use patterns. Each ICD-10 group was used as a predictor variable, and each PS was used as a separate dependent variable. Significant findings were passed into the multivariable logistic regression model (Table 6).

**Table 6:** Results of multivariable logistic regression. Risk of disorders occurrence according to PS use.

	*p*	*Exp*(*B*)	*CI l–h*
*F00–F99*			
PS	0.4	1.285	0.713–2.316
BDZ	0.005	3.83	1.48–9.85
F00–F09*			
BDZ	< 0.001	4.72	2.09–10.64
F20–F29*			
THC	0.017	3.253	1.23–8.56
COC	0.103	3.922	0.76–20.23
F30–F39*			
BDZ	0.02	6.325	1.34–29.88
F40–F49*			
BDZ	0.026	5.792	1.24–27.11
F70–F79*			
PS	0.024	3.975	1.2–13.17

Subsequently, cross tabulations were performed to examine healthcare-related factors concerning PS use, consequently both overall and specific. These factors included the frequency of hospitalization as indicated by the repetition of the PESEL number in the database, the type of admission (emergency versus elective), and the history of psychiatric treatment (Table 7). Regression analyses were then conducted with the hypothesis that PS use would increase the likelihood of these healthcare-related factors being observed. Bivariate logistic regression analysis enabled the identification of associations between PS use (dependent variable) and the healthcare-related factors (predictors) (Table 8). Significant predictors identified through bivariate analysis were then included in a multivariable regression model to assess their collective impact on PS use (Table 9).

**Table 7:** The relationship between declared lifetime psychoactive substance use and seeking psychiatric treatment.

	Total		No PS		PS		ATS		Cannabis		BDZ		NPS		OPI		COC	
	N	%	N	% of PS	N	% of PS	N	% of ATS	N	% of THC	N	% of BDZ	N	% of NPS	N	% of OPI	N	% of COC
*Hospitalizations*																		
Single	735	72.4	621	74.3	114	63.7	44	67.7	72	62.6	17	56.7	17	58.6	16	66.7	10	62.5
Multiple	280	27.6	215	25.7	65	36.3	21	32.3	43	37.4	13	43.3	12	41.4	8	33.3	6	37.5
*Mode of admission*																		
Emergency	366	36.1	279	33.4	87	48.6	31	47.7	61	53.0	13	43.3	15	51.7	13	54.2	6	37.5
Elective	649	63.9	557	66.6	92	51.4	34	52.3	54	47.0	17	56.7	14	48.3	11	45.8	10	62.5
*Previous psychiatric treatment*																		
No	296	29.2	263	31.5	33	8.5	12	20	22	19	2	6.7	4	14	2	8.3	5	31.2
Yes	719	70.8	573	68.5	146	81.5	52	80	93	81	28	93.3	25	86	22	91.7	11	68.8

**Table 8:** Results of bivariate logistic regression conducted to assess the linkage between seeking psychiatric treatment factors and SA use.

Seeking psychiatric treatment factor	PS			ATS			Cannabis			BDZ			NPS			OPI			COC		
	*p*	*Exp*(*B*)	*C.I. l–h*	*p*	*Exp*(*B*)	*C.I. l–h*	*p*	*Exp*(*B*)	*C.I. l–h*	*p*	*Exp*(*B*)	*C.I. l–h*	*p*	*Exp*(*B*)	*C.I. l–h*	*p*	*Exp*(*B*)	*C.I. l–h*	*p*	*Exp*(*B*)	*C.I. l–h*
Multiple hospitalizations	0.002	1.74	1.22–2.48	0.074	1.63	0.95–2.81	0.013	1.61	1.11–2.51	0.055	2.06	0.99–4.29	0.097	1.81	0.89–4.01	0.145	1.96	0.79–4.64	0.179	2.05	0.724–5.6
Emergency vs Elective admission to hospital	< 0.001	1.88	1.36–2.62	0.96	1.01	0.64–1.59	< 0.001	2.24	1.49–3.26	0.401	1.39	0.66–2.85	0.08	1.98	0.93–4.06	0.067	2.14	0.95–4.82	0.73	1.26	0.38–3.94
Previous psychiatric treatment	0.001	2.03	1.35–3.05	0.096	1.69	0.91–3.17	0.013	1.85	1.14–3.01	0.015	5.97	1.41–25.17	0.075	2.63	0.91–7.62	0.039	4.64	1.08–19.86	0.46	0.85	0.61–1.25

The regression model was run separately for each factor as dependent variable. PS use variables were sequentially entered as predictors. Significant results passed into the subsequent multivariable logistic regression model (Table 9).

**Table 9:** Results of multivariable logistic regression, conducted to assess the linkage between seeking psychiatric treatment factors and SA use.

Substance	*p*	*Exp*(*B*)	C.I. l–h
*Multiple hospitalizations*			
PS	0.132	1.513	0.88–2.59
Cannabis	0.688	1.14	0.6–2.16
*Emergency versus elective admission to hospital*			
PS	0.239	1.366	0.81–2.3
Cannabis	0.112	1.651	0.89–3.07
*Previous psychiatric treatment*			
PS	0.611	1.227	0.56–2.7
Cannabis	0.436	1.421	0.58–3.44
BDZ	0.071	4.284	0.88–20.8
OPI	0.177	2.918	0.62–13.81

Adjusted odds ratio (OR) expressed in Exp(B), confidence interval, and p-values were presented in the logistics regression.

### Ethics

The authors declare that the study complies with the ethical guidelines and applicable local law. The study was designed as a retrospective, cross-sectional review of medical data, therefore consent to participate was not required.

This study was approved by the local Bioethics Committee of the University of Warmia and Mazury in Olsztyn—Decision No. 14/2020 on April 29, 2020. The informed consent was waived by The Bioethics Committee of the University of Warmia and Mazury in Olsztyn. Data were introduced into an Excel spreadsheet using a coded ID number, which could not be used to retrospectively identify individual patients. The spreadsheet was password-protected and stored on the University servers only. The password was available only to the authors of the study.

## Results

There were 3505 admissions to the hospital during the study period, 1525 of which included patients diagnosed with mental and behavioural disorders due to the use of alcohol (Section F10 of ICD10). Cases with incomplete medical documentation were excluded from the study group. In the case of patients who were hospitalized more than once, second and subsequent hospitalizations were also not included. The remaining 1015 records of patients were included in the study.

### Demographic data of the study sample

**Figure 1:**
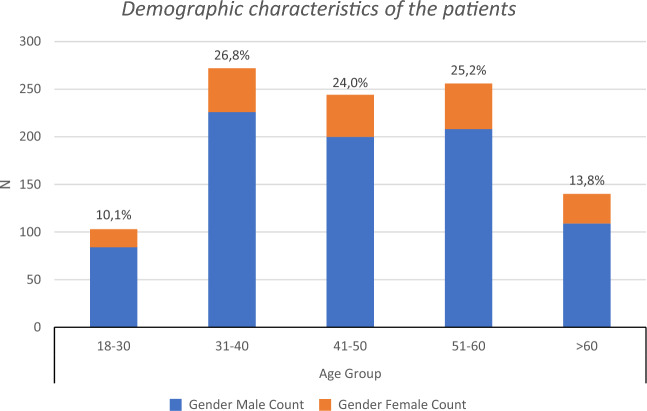
Demographic data of the study sample. The study comprised a total of 1,015 participants, with a distribution of 827 males (81.5%), and 188 females (18.5%).

Figure 1 presents the demographic data of the study sample. Out of 1015 cases, 827 were men (81.5%), and 188 were women (18.5%).

In the examined group, significant sex distribution differences were observed (*χ*^*2*^ = 402.29; *p* < 0.001), favoring males. The median age was 45 years, and a mean of 46 years. The group was categorized by age, with the 31–40 age group being the most represented (26.8%, N = 272) and the 18–30 age group the least (10.1%, N = 103).

#### Prevalence and types of psychoactive substances declared in the examined population

**Figure 2:**
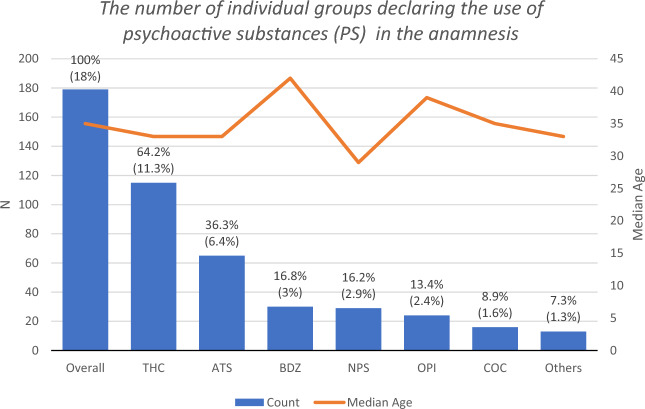
Types of psychoactive substances which were declared in anamnesis, combined with the median age of PS users. Values represent the percent of specific substance users, thus, overall substance users (N = 179) equal 100%. Values in braces represent the percent of substance users in all study participants (N = 1015).

179 individuals (17.6%) declared taking PS at least once in their lifetime, including 153 males and 26 females. Among these, 112 individuals reported using only one type of PS, whereas 67 individuals declared using more than one type (62.6%, and 37.4% of those reporting PS use, respectively). It should be specified that the group of people declaring taking more than one substance includes people declaring taking two, three and more types of PS during their lives. The difference between the number of individuals declaring the use of a single type of PS and those using multiple types was statistically significant (*χ*^*2*^ = 11.31, *p* = 0.0008), favoring single substance users. Figure 2 presents the types of psychoactive substances which were declared in anamnesis.

The median and mean age of the individuals reporting the use of specific types of PS differed; however, these differences were not significant (*χ*^*2*^ = 18.378, *p* = 0.243), possibly due to the sample size of the age subgroups.

Participants who reported lifetime use of any PS, along with the six subgroups reporting lifetime use of specific substances (cannabis, ATS, BDZ, NPS, OPI, COC), were further analyzed. Noteworthy, “others” group included individual cases of people declaring taking various substances, e.g. Z-drugs, LSD, hallucinogenic mushrooms and others. Due to the heterogeneity of this group, it was not included in further analyses.

Table 1 presents detailed data of PS use, including both overall prevalence and specific substance types, across age groups and gender. Due to the fact that some participants declared taking more than 1 (2, 3, etc.) types of substances, the numbers shown in the table are not the same as the absolute size of the study group.

### Associations between PS use, age group and gender

Bivariate logistic showed a significant association between PS use and age group. Specifically, males aged 18–30 and 31–40 demonstrated increased OR for PS use, whereas those aged 51–60 and over 60 showed decreased OR. In contrast, the only significant finding for females was within the 51–60 age group, with a decreased OR of 0.3 (*p* = 0.045).

For ATS users, similar age-related trends were observed among males, except for the 51–60 age group, where no significance was observed. No significant associations were detected for females in this group.

Cannabis use among males showed significant associations in all age groups except for 41–50, with increased ORs for those aged 31–40 and younger, and decreased ORs for ages 51–60 and above. Female cannabis users did not exhibit significant associations.

NPS use among males was significantly associated with the 18–30 and 31–40 age groups, except for the 41–50 age group, which showed decreased odds of use.

OPI use presented a significant association for males in the 31–40 age group only.

BDZ use highlighted a notable increase in ORs for females over 60, showing a 3.8 OR increase.

COC use among males was significantly associated with the 18–30 and 31–40 age groups.

All reported associations were statistically significant with *p* < 0.05.

Further analysis using multivariable logistic regression resulted as follows: associations between general PS use and age groups were reproduced from bivariate regression results. Three male age groups of cannabis users (18–30, 31–40 and 51–60) were found to be significantly linked to cannabis use, with increased odds present in age groups from 18 to 30 and 31–40. In older participants, decreased odds were observed. No significant associations were found for female cannabis users. Regarding ATS users, only the associations between ATS use and male 21–30 and 31–40 age groups were found to be significant, exhibiting increased odds. A similar pattern was observed in NPS users. Regarding the BDZ, only female > 60 group users presented significant association. Falling into the male 31–40 age group increased the odds of COC or OPI usage, moreover male 18–30 group increased the odds of COC usage.

All of the above observations were significant at *p* < 0.05.

Detailed results of bivariate and multivariable logistic regression are shown in Tables 2 and 3 accordingly.

### Comorbid diagnoses and their association with PS use

Disorders within the F10 group were diagnosed in 890 individuals. Additionally, the remaining 119 individuals (11.7%) were diagnosed with psychiatric disorders, including 28 cases from the F11–F19 group. This diagnoses group was excluded from further analysis, since it consists of drug use disorders, and occurred exclusively in individuals who reported using PS. Among the individuals who reported using PS (N = 179), additional psychiatric disorders were diagnosed in 52 people. Among these cases, the coexistence of F11–F19 diagnoses was found in 28 individuals, while other disorders were present in 24 cases. Table 4 shows the distribution of additional diagnoses.

Bivariate logistic regression showed a significant association between declared psychoactive substance lifetime use and general psychiatric comorbidity (excluding the F10–F19 group) in the study cohort. Specifically, using PS increased OR of F70–79 disorders.

In the case of a group that declared lifetime use of cannabis, only a significantly increased OR of F20–F29 occurrence was found. The same situation was observed in a group that declared a lifetime of COC. Declared lifetime use of benzodiazepines increased the OR of general psychiatric comorbidity and also disorders within the F00–09, F30–F39, and F40–F49 groups.

The multivariable regression analysis revealed the following significant associations: BDZ use was associated with increased OR of general mental and behavioral disorders occurrence (F00–F99), particularly within the F00–F09, F30–F39 and F40–F49 groups. Cannabis use was linked to a higher OR of F20–F29 disorders occurrence. Furthermore, the use of any PS was associated with increased OR of F70–F79 group disorders. Detailed results of these associations are presented in Table 8.

### Seeking psychiatric treatment and its association with PS use

Table 7 shows the relationship between declared lifetime psychoactive substance use and the frequency of hospitalizations in the examined period based on the PESEL number, the mode of admission, and data from the anamnesis regarding previous psychiatric treatment (both outpatient and inpatient treatment). The length of hospitalization was not assessed because in Poland it results from contract procedures with the national insurer.

Bivariate logistic regression analysis demonstrated a significant linkage of psychoactive substance use to all of the above factors, i.e. PS use significantly increased the OR of all the factors. Regarding specific substance use, observed significant associations were as follows: for cannabis, all the factors were significant. In ATS users, no significant linkage was found. For BDZ and OPI users, former psychiatric treatments were significant.

Subsequent multivariable logistic regression yielded no significant results, therefore, bivariate regression results were further discussed.

## Discussion

According to the European Monitoring Centre for Drugs and Drug Addiction (EMCDDA), nearly a quarter of the adult population (aged 15–64) in the European Union has experimented with illegal substances at some point in their lives. The most commonly used drugs are cannabis, ATS (3,4-methylenedioxy-methamphetamine (MDMA) and amphetamine), cocaine and opioids^15^.

In Poland, the frequency of taking PS, depending on the sources, is estimated at 6.3%^3^ to 16.4% of respondents^15^.

The results of our research showed that in the entire study group, only 17.6% declared using PS in their lives, which stands for a lower percentage than the EMCDDA data and is similar to the data from Poland.

We also observed differences in the frequency of the most popular declared substances compared to EMCDDA data. In our study, the most frequently declared intake was: cannabis (11.3%), next ATS (6.4%), BDZ (3%), NPS (2.9%), OPI (2.4%). Only 1.6% of people were reported taking cocaine. This situation may be a country-specific phenomenon. Cocaine is a relatively unpopular drug in Poland compared to the other types of drugs^2,3,16,17^.

Another large Polish survey study, PolDrugs, which was conducted among Internet users and concerned the recreational use of psychoactive substances, showed that the most frequently declared substances were cannabis, MDMA (ATS group) and hallucinogens. The results of this study differ from ours, which may be due to the different structure of the study group (patients of a psychiatric hospital in a given year in our case, in PolDrugs—Internet users and respondents)^18^.

As we expected, the prevalence of taking psychoactive substances was the highest among the youngest individuals and gradually decreased with age. Additionally, the majority of individuals who reported using psychoactive substances were males, both overall and within specific age groups. These findings align with what is generally reported in the literature^2,15^. The only group that stood out was the one reporting the use of BDZ, in which the percentage of females increased with age. In the 41–50 age group and beyond, women outnumbered men, and in the oldest group, this difference was statistically significant in the logistic regressions.

Comorbid psychiatric diagnoses (other than AUD or DUD) accounted for a total of 9% of people in the study group. In the group of people declaring not taking psychoactive substances, this percentage was 8%, while in the group of people declaring lifetime PS, it was 13.4%. Lifetime use of psychoactive substances significantly increases the odds of illnesses (1.77 in bivariate logistic regression). The comorbidity of psychiatric disorders in our material is similar to Morandi’s results^9^ and much lower than in other studies^4,8,10–12^. On the other hand, 68.5% of individuals who denied using PS declared previous psychiatric treatment, while 81.5% of those who admitted lifetime use of PS reported the same. These findings align with literature indicating that over 75% of individuals with AUD have received mental health treatment in the past^13^.

Therefore, our study revealed a large difference between the frequency of mental disorders and the frequency of psychiatric treatment declared in the past. This difference may be attributed to the fact that the study focused on individuals hospitalized for AUD and comorbid mental illnesses diagnosed at the time when specific symptoms were reported, which does not rule out the presence of such issues in the past. The work of Morandi, which examined a similar group of patients—individuals treated in an alcohol addiction center who were diagnosed with other psychiatric disorders, may account for results similar to those found in our study^9^. However, other cited works differed methodologically, as they focused on groups of patients with specific psychiatric diagnoses and alcohol or drug use disorders^4,8,10–12^. Despite these methodological differences, it remains evident that individuals with AUD and concurrently using psychoactive substances exhibit a higher incidence of psychiatric disorders.

Looking at specific substances, the highest percentage of illnesses was found in people who reported taking BDZ, followed by COC.

The highest percentage of mental disorders in the group of people who declared taking BDZ may be because this means the age of the patients was the highest. Benzodiazepines, however, are a relatively popular group of drugs abused in primary care in Poland. It is usually observed, that along with the ageing process, the frequency of benzodiazepine use increases^19,20^. Therefore, it is possible that despite the clear indication of non-therapeutic reasons for taking PS when collecting interview data, some respondents may have made a mistake. Hence, data regarding benzodiazepines should be treated with particular caution in this case. We also found a relationship between cannabis use and the occurrence of disorders from the F20–F29 group, which is well-documented in the literature^6,7,21,22^. The literature also provides data on the relationship between COC use and the occurrence of disorders from the F20–F29 group^22^.

Referring to the discussion of the second aim, i.e. seeking psychiatric treatment: the results of bivariate logistic regression analysis demonstrated that lifetime use of PS significantly increased the likelihood of all factors occurring. The same situation occurred in the case of people declaring lifetime use of cannabis. In the case of the remaining subgroups, a statistically significant increase in the probability was demonstrated only in the group of BDZ users and in the group of OPI users for previous psychiatric treatment.

Explaining such outcomes is challenging; however, it might be related to age in the case of the BDZ users group. These individuals were the oldest (relatively older compared to other users), which logically increases the probability of having undergone previous psychiatric treatment. We believe that the absence of statistically significant connections for the other groups likely stemmed from the limited size of the sample.

The effect of cannabis on the frequency of hospitalizations was studied, among others, by Gryczynski et al. on a very large group of respondents. His analysis showed that the odds of hospitalization were 16% points lower for people who used cannabis but did not meet diagnostic criteria for substance use disorder, compared to abstainers. However, in the case of people with cannabis use disorders, the odds of hospitalization were similar to abstainers^23^.

Other publications showed that the use of cannabis was associated, among others with greater frequencies of the risk of acute healthcare use^6^.

Unfortunately, we couldn’t find any literature specifically on psychiatric hospitalizations alone, despite these data being related to general hospitalizations. When comparing our findings with the available data, it was evident that in the case of AUD, unlike in individuals without AUD, even lifetime use of cannabis increased the frequency of psychiatric hospitalizations within a year. Cannabis, indicated as a potential medicine for many diseases, shows a strong correlation with seeking psychiatric treatment in the study group, so it has a clearly negative impact on health.

Taken together, a greater need to seek treatment indicates a more severe course of the disorder. Previous research has pointed out that the coexistence of AUD and DUD causes greater psychopathology and severity of AUD relative to those in the AUD-only group^4^. Based on our data, we can broaden the above observations by mentioning that even lifetime use of PS by people with AUD is connected with a more severe course of the underlying disorder. It’s important to highlight that cannabis, despite being suggested as a potential treatment for various diseases^6,7^, demonstrates a significant association with the pursuit of psychiatric treatment within the study group, indicating a clearly negative effect on health.

The strong point of the study is the relatively numerous and well-defined group (1015 patients of the psychiatric hospital, hospitalized during the year).

The small sample sizes of certain subgroups and methodological variations, such as different disorder classifications (ICD-10, DSM-IV), could account for the disparities between the data presented in this paper and the information provided in the literature.

Another weak point of the study is the way these data were collected. It did not allow for a more precise assessment of, e.g. risk factors (serious risky behavior, upbringing in an incomplete family, educational problems etc.) or psychosocial consequences of using psychoactive substances.

Considering the time that has passed since the research, legislative changes introduced over time and new trends in using substances, all those factors may limit the patterns of use. Perhaps in current studies the structure of the PS might be slightly different.

The retrospective analysis of medical records due to any patients’ highly individual psychiatric documentation did not give a chance for a very thorough study of separate factors.

## Conclusion

Our findings have shown the significant prevalence of the phenomenon of concurrent use of alcohol with other drugs in the population of people with disorders due to the use of alcohol.

People who concurrently use alcohol with other drugs significantly more often suffer from psychiatric comorbidities than people who deny the use of other drugs.

These individuals are significantly more likely to seek psychiatric treatment than patients who deny taking psychoactive substances. In conclusion, there is a clear need for further research to understand the etiology and develop effective prevention and intervention measures.

## Data Availability

The datasets analyzed during the current study are not publicly available due to they contain sensitive data theoretically allowing patient identification, but are available from the corresponding author on reasonable request.
